# Trend analysis of journal metrics: a new academic library service?

**DOI:** 10.5195/jmla.2017.98

**Published:** 2017-07-01

**Authors:** Peter Kokol

## Abstract

**Objective:**

Temporal trends in source normalized impact per paper (SNIP) values for the three top-ranking nursing journals were analyzed and compared to explore whether predicting future SNIP values based on trend analysis could be an innovative service provided by librarians.

**Methods:**

The *International Journal of Nursing Studies, Journal of Nursing Scholarship,* and *Journal of Advanced Nursing* were the three top-ranked nursing journals according to 2015 SNIP values. SNIP values for the selected journals were retrieved from the Scopus database, and extracted data were exported to Joinpoint trend analysis software to perform trend analysis.

**Results:**

The trend in SNIP values for the *International Journal of Nursing Studies* was the most stable and positive, whereas the trend in SNIP values for the *Journal of Advanced Nursing* was the most negative. The annual percentage change of the most recent trend line, which is the best indicator for predicting future SNIP values, was the largest for the *International Journal of Nursing Studies.*

**Conclusions:**

Predictions of journal metrics based on statistical joinpoint regression may not be completely accurate. Using this technique, however, a librarian can reasonably claim which journal will retain or even improve its prestige in the future and thus safely advise prospective authors on where to publish their research.

## INTRODUCTION

The rapid development in information and communication technology has triggered a need for librarians to constantly adapt to the requirements of ever-changing information environments. Bibliometrics is an emerging field in information science, and its increasing importance provides opportunities for librarians to develop and provide innovative services and thus aid their customers in an academic environment more effectively [1]. Additionally, the increasing importance of research assessment based on bibliometric indicators in acquiring project funding, academic institution rankings, career development, and institutional promotion is prompting academic institutions to measure these indicators [2, 3]. Familiarity with bibliographic databases and expertise in collecting, categorizing, and analyzing data qualifies librarians to meet these challenges [4].

Research success assessment relies strongly on the status of the journals in which the research is published [5]. The impact factor is frequently considered the main indicator of a journal’s status. However, a problem with the impact factor is that it is calculated with past data; hence, its value reflects the prestige of a journal one or two years before the current date. The duration of the peer-review and publishing process can make this time lag even longer. Consequently, authors do not know what the actual value of an impact factor will be at the time when their paper is published. Therefore, analyzing trends in journal metrics and predicting their future values could be an innovative service provided by academic librarians.

In this study, temporal trends in source normalized impact per paper (SNIP) values [6] for the three top-ranking nursing journals were analyzed and compared. SNIP was selected over the more familiar impact factor for two reasons. First, SNIP values were assigned to the current top-ranking nursing journals starting in 1999, whereas impact factors were assigned to some of these journals only after 2006. Second, SNIP measures contextual citation impact, which takes into account the citation potential of the field in question. That is, the SNIP is calculated as the ratio of the journal’s average citation count per publication to the average number of references in reference lists of citing papers [6].

## METHODS

The *International Journal of Nursing Studies (IJNS), Journal of Nursing Scholarship (JNS),* and *Journal of Advanced Nursing (JAN)* were the 3 top-ranked journals in the “Nursing (all)” category according to SNIP in 2015. SNIP values for the 3 selected journals for the period 1999–2015 were retrieved from the Scopus database (Elsevier, Leiden, Netherlands) using the Scopus Analyze and Export services. Extracted data were exported to Joinpoint (JP) trend analysis software, version 4.4.0.0, open license software that is available from the National Cancer Institute website [7]. A joinpoint is a point at which a significant change in trend occurs. For each joinpoint, annual percent change (APC) is estimated using regression analysis, with calendar year as a predictor [8]. JP software analyzes trend data and derives the simplest joinpoint model according to a user-supplied number of joinpoints (minimal and maximal number of joinpoints). JP software then uses a series of permutation tests to compute the number of joinpoints to best fit the data. Trend models were created for each journal separately. After experimenting with different numbers of jointpoints, the models with 3 joinpoints provided the best prediction accuracy. The APC for the last trend segment was used for prediction purposes.

The models were validated by training them on SNIP data for the period 1999–2014, using the models to predict SNIP values for 2015, and then comparing these values to the actual SNIP values in 2015. After validation, models were built on SNIP data for the entire period 1999–2015 and used to estimate SNIP values for 2016 and 2017.

## RESULTS

**Figure 1:**
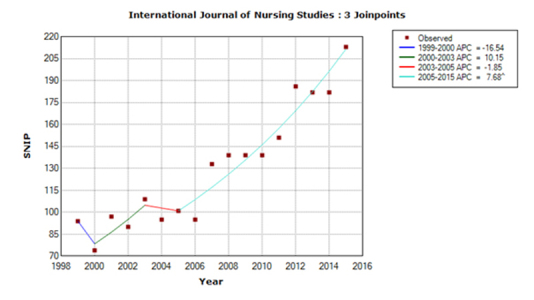
Trend model for the *International Journal of Nursing Studies (IJNS)* SNIP values in the y-axis are shown without a decimal point (e.g., a SNIP value of 1.00 is represented as 100).

**Figure 2:**
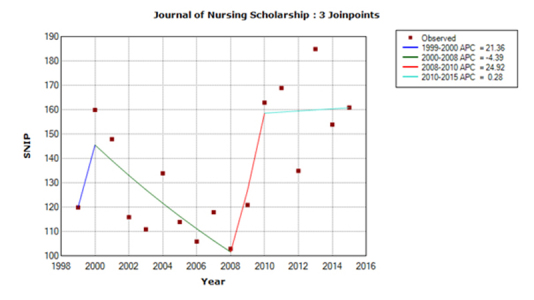
Trend model for the *Journal of Nursing Scholarship (JNS)* SNIP values in the y-axis are shown without a decimal point (e.g., a SNIP value of 1.00 is represented as 100).

**Figure 3:**
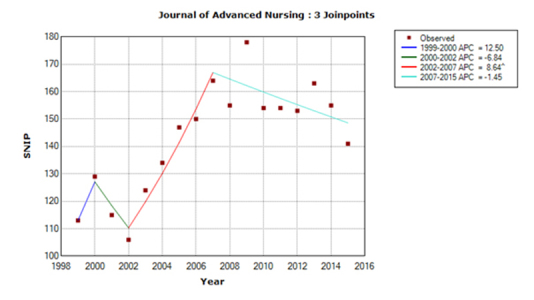
Trend model for the *Journal of Advanced Nursing (JAN)* SNIP values in the y-axis are shown without a decimal point (e.g., a SNIP value of 1.00 is represented as 100).

For *IJNS,* the predicted SNIP value for 2015 was 1.89, based on an APC of 3.62 for the last trend line during 1999–2014, whereas the actual SNIP value for 2015 was 2.13. For *JNS,* the predicted SNIP value was 1.51, based on an APC of –1.71, whereas the actual SNIP value was 1.61. For *JAN,* the predicted SNIP value was 1.58, based on an APC of 2.21, whereas the actual SNIP value was 1.41. The accuracy of predictions was 89% for *JAN* and *IJNS* and 94% for *JNS.* The trend models for 1999–2015 are shown in Figures 1 to 3. All three journal’s SNIP trends were explained by three joinpoints, the SNIP trend dynamics differed substantially between journals.

The *IJNS* SNIP trend was the most stable of the 3 journals, with just 2 slightly negative trend lines (Figure 1). The APC for the last segment was just below 8%, suggesting that the SNIP value will probably increase by 8% yearly in the near future.

The *JNS* SNIP trend had only one negative trend line (Figure 2), although its slope was much steeper than those of *IJNS.* Additionally, the duration of this negative trend was much longer (i.e., nine years). This negative trend was followed by a very steep rise, which was interrupted in 2010 with a still positive but more modest positive trend. Due to these large fluctuations, predictions of SNIP values cannot be as accurate as those for *IJNS.* The very low APC for the last five years suggests that SNIP values may stay in the range of its 2015 value in the near future.

With 2 positive and 2 negative trend lines characterized by very different APCs, the SNIP dynamics for *JAN* was the most chaotic (Figure 3), which might make predictions quite inaccurate. However, the APC of the last segment was –1.45%, suggesting that the SNIP value will continue to fall between 1% and 2% yearly in the near future.

## DISCUSSION

The trend models presented here can enable prediction of future trends in SNIP values by taking into account various factors such as the stability of the trends, the number of negative and positive trend lines, and the magnitude of APCs. The predictions for SNIP values for 2016 (to be published in mid-2017) and 2017 (to be published in mid-2018) are as follows:

- *IJNS:* 2.29 for 2016 and 2.46 for 2017
- *JNS:* 1.61 for 2016 and 1.62 for 2017
- *JAN:* 1.39 for 2016 and 1.37 for 2017

These values are only predictions based on statistical joinpoint regression. However, a librarian could reasonably claim that the *IJNS* is the safest option for prospective authors to publish their research, as its SNIP value will probably increase and the journal will likely retain its prestigious status in the future.

## References

[1] Gumpenberger C, Wieland M, Gorraiz J (2012). Bibliometric practices and activities at the University of Vienna. Libr Manag.

[2] Brandon AN (1963). Publish or perish. Bull Med Libr Assoc.

[3] Baumann H (2002). Publish and perish? the impact of citation indexing on the development of new fields of environmental research. J Industrial Ecol.

[4] Bladek M (2014). Bibilometrics services and the academic library: meeting the emerging needs of the campus community. Coll Undergrad Libr.

[5] Moed HF (2010). Measuring contextual citation impact of scientific journals. J Informetrics.

[6] Kim HJ, Fay MP, Feuer EJ, Midthune DN (2000). Permutation tests for joinpoint regression with applications to cancer rates. Stat Med.

[7] National Cancer Institute (2016). Joinpoint regression program. Version 4.3.1.0.

[8] Petersohn S (2016). Professional competencies and jurisdictional claims in evaluative bibliometrics: the educational mandate of academic librarians. Educ Inf.

