# Correction

**DOI:** 10.5195/jmla.2018.412

**Published:** 2018-01-02

**VOLUME 105**

**105(3) July, page 242**

Kokol P. Trend analysis of journal metrics: a new academic library service? J Med Libr Assoc. 2017 Jul;105(3):240–2. DOI: http://dx.doi.org/10.5195/jmla.2017.98.

Figure 3 in the portable document format (PDF) and print versions is a duplication of Figure 1. The figure should be:

**Figure 3:**
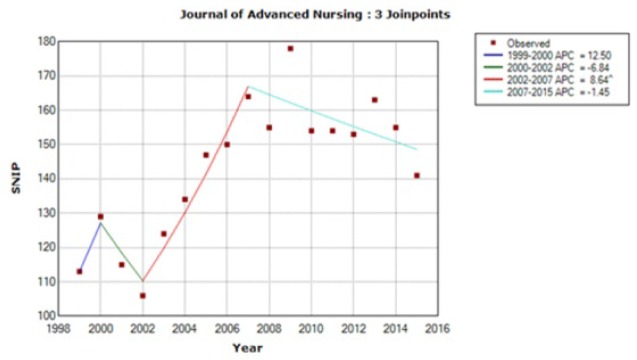
Trend model for the *Journal of Advanced Nursing (JAN)* SNIP values in the y-axis are shown without a decimal point (e.g., a SNIP value of 1.00 is represented as 100).

